# Pain in People With Fibromyalgia Syndrome (FMS) Undergoing or Following Surgery: A Systematic Narrative Review

**DOI:** 10.1155/prm/2352060

**Published:** 2026-01-22

**Authors:** Richard J. Berwick, Sara Siew, Sarah Curtis, Michelle Maden, Ruaraidh Hill, Andreas Goebel

**Affiliations:** ^1^ Institute of Life Course and Medical Sciences, University of Liverpool, Liverpool, UK, liv.ac.uk; ^2^ Department of Pain Medicine, The Walton Centre NHS Foundation Trust, Longmore Lane, Liverpool, UK, thewaltoncentre.nhs.uk; ^3^ Department of Anaesthesia, Liverpool University Hospitals NHS Foundation Trust, Liverpool, UK; ^4^ Institute of Population Health, University of Liverpool, Liverpool, UK, liv.ac.uk

**Keywords:** chronic pain, fibromyalgia syndrome, FMS, postoperative pain, surgery, surgical outcome

## Abstract

**Background:**

Fibromyalgia syndrome (FMS) is a chronic pain condition characterised by widespread pain, fatigue, sleep disturbance and cognitive problems. This systematic review aimed to characterise the perioperative experiences of FMS patients undergoing elective surgery, including pain, local anaesthetic (LA) efficacy, complications, and functional outcomes postoperatively.

**Methods:**

This systematic review was reported in accordance with the PRISMA guidelines. The protocol was registered on PROSPERO (CRD: 42022309297). MEDLINE, Embase and CENTRAL were searched from 1990 to 28 June 2024. Two reviewers independently undertook screening, data extraction and quality assessment. A narrative synthesis was conducted.

**Results:**

Nineteen relevant studies published between 1999 and 2024 were identified, mostly focussing on orthopaedic surgeries. Evidence suggests FMS patients may experience more acute postoperative pain at the surgical site and increased widespread spontaneous pain compared to non‐FMS individuals. Opioid requirements may also be greater and is linked to the extent of symptomatology. Little data existed on the benefit of LA techniques. Large cohort studies found no overall increase in complications, but within FMS patients undergoing orthopaedic surgery, there were increased 30‐ and 90‐day complications including anaemia, readmissions and pneumonia. Data indicate poorer early postoperative functional recovery in FMS patients. Interestingly, some studies reveal that treating perpetuating visceral pain sources may lead to improvements in spontaneous FMS pain and sensory thresholds beyond 3 months.

**Conclusions:**

The review highlights a paucity of evidence characterising the immediate perioperative course in FMS patients, indicating an important area for future research to optimise patient experiences and outcomes; however, individualised patient discussions are key.

## 1. Introduction

Fibromyalgia syndrome (FMS) is a chronic widespread pain condition affecting 2%–4% of the population [1, 2]. The condition is characterised by pain and a constellation of other symptoms, most markedly fatigue, sleep disturbance and cognitive problems [3, 4]. Its aetiology remains uncertain but abnormal nociceptor function [5–7] and central amplification may contribute to the FMS phenotype [3, 8–10]. This type of altered function, in the absence of clear evidence of tissue damage or somatosensory disease, is defined by the IASP as nociplastic pain [11]. In addition, however, there may also be elements of peripheral small fibre neuropathy [12]. Recent evidence suggests that there is an autoimmune component with peripherally binding pro‐analgesic IgG autoantibodies [13–15] and other immune abnormalities [16, 17] which may all lead to an increased peripheral nociception [18].

The recent Royal College of Physician Guidance on the diagnosis of fibromyalgia [19], which covers both initial diagnosis and subsequent interactions, emphasises robust communication and information giving. One particularly important consideration for ongoing care of these patients is surgery, which typically involves a noxious insult to the body. Nociceptors in fibromyalgia are hyperactive [20, 21], and increased sensitivity in patients [10, 19, 22–25] can be expected to lead to different experiences during, and following, this noxious surgical insult, compared to nonsensitised individuals. Increased hypersensitivity is clear even in the absence of deficient descending inhibition [24]. It is possible, therefore, that postoperative pain is a significant problem for patients with FMS. Indeed, surgical incision is more painful for FMS individuals [26], and even in non‐FMS states, such as isolated knee osteoarthritis [27], preoperative mechanical hypersensitivity predicts the intensity of postoperative pain.

**Figure 1:**
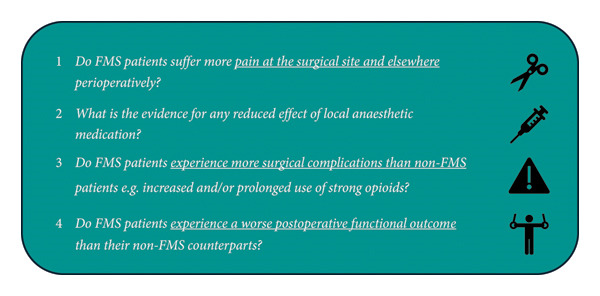
Key questions. Questions identified from the patient public engagement activities as part of guideline development.

During a patient engagement activity conducted for the UK FMS diagnostic guidelines development process (January 2021), participating FMS patients reported difficult perioperative incidents. Several key questions emerged, for which there was limited information. These are summarised in Figure 1. Firstly, do FMS patients suffer more pain perioperatively at the surgical site and elsewhere? Given anecdotal reports from our patient group of local anaesthetic (LA) being partially inefficacious, secondly, do usual anaesthetic techniques, involving LA compounds, work within the expected time periods and do they then have the expected effect? Thirdly, do FMS patients experience more complications from their operative management than non‐FMS patients? Fourthly, do FMS patients experience worse early postoperative functional outcomes than their non‐FMS counterparts?

We thus conducted a narrative systematic review, aiming to provide evidence to inform anaesthetic and surgical practice, with ultimate goals of improving perioperative patient experience regarding pain, and postoperative complications. Recent reviews inspecting postoperative outcomes in patients with FMS have generally found worse longer‐term (beyond 6 months) outcomes in orthopaedics, in terms of both pain and complications [28–30]. We have, therefore, used a broad search strategy to include patients with FMS undergoing all types of noncancer surgery, focussing, primarily, on the perioperative time course, in this thorough, up‐to‐date systematic review of the literature.

## 2. Methods

This systematic review is reported in accordance with the Preferred Reporting Items for Systematic re‐views and Meta‐Analyses (PRISMA). The protocol for this review is registered on PROSPERO as CRD: 42022309297.

### 2.1. Search Strategy

MEDLINE, Embase and CENTRAL were searched from 1990 to 28 June 2024. Search terms were developed iteratively and approved by the research team. Searches were conducted by M.M. (see supporting information Figure S1 for the search strategy). Key concepts searched were Fibromyalgia AND (perioperative/postoperative OR postoperative pain/complications). Searches were restricted to English language publications. Search results were combined, and duplicates removed by S.C. and S.S. Reference lists of the included studies were read to identify additional relevant studies and systematic reviews.

### 2.2. Inclusion and Exclusion Criteria

Our population comprised people with FMS diagnosed medically undergoing elective surgery. This could be according to the relevant American College of Rheumatology (ACR) criteria [4, 31–33]. However, for some database studies, means of diagnosis was not included. It was sufficient if diagnosis was record‐linked. We included any study designs, including randomised or nonrandomised controlled trials, retrospective or prospective cohort studies, case control studies, case series (reporting on five or more participants) and retrospective registry analyses.

We excluded studies reporting on children or young people aged 17 years or younger and those reporting on nonelective surgery or surgery for malignancies. We excluded nonempirical research publications such as editorials or commentaries, literature reviews and systematic reviews.

**Figure 2:**
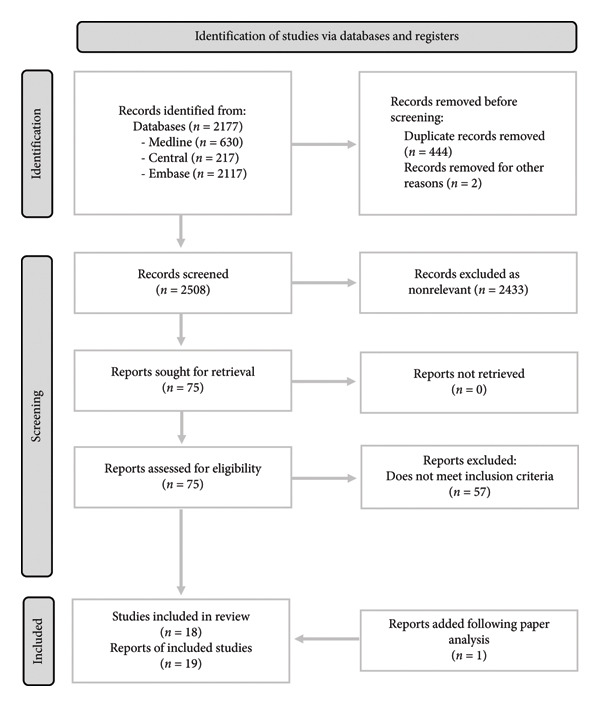
PRISMA flow chart. Study selection process details as per PRISMA guidance [54].

All article titles and abstracts were then screened by two reviewers (R.B., S.C. or S.S.) independently, in a two‐stage process, and articles meeting the inclusion criteria were selected for full‐text analysis. Irrelevant articles were then removed following screening, and a shortlist of articles was compiled for full‐text eligibility analysis. Full texts were obtained, and S.C. and S.S. decided whether to include or exclude articles via consensus with input from senior author A.G. The selection and screening process is detailed in Figure 2.

### 2.3. Data Extraction and Quality Assessment

Study characteristics, methodology data and results from selected full‐text studies were extracted independently by R.B. and S.S. The main outcomes included postoperative pain measured by a validated scale, such as numeric rating scale (NRS) or visual analogue scales (VAS), at operation site or elsewhere, including overall pain intensity, widespread pain index (WPI) and symptom severity scales (SSS), such as used in the 2016 ACR criteria [4, 31–33]. We also sought to interrogate the use of medication to manage pain preceding or postsurgery. Postoperative function (such as range and ease of movement or mobility), duration of hospital stays, patient experience or satisfaction and quality of life measures (such as EQ‐5D, SF‐36) were also extracted. We limited our data to findings within the 6 months.

Data were abstracted into a pretested Microsoft Excel‐based form. Data relating to both study design, outcomes/findings and quality were extracted by one reviewer (R.B.) and independently checked for accuracy by a second reviewer (S.S.).

The articles were assessed for bias to address their external and internal validity. Independently, R.B. and S.S. appraised each article with The Newcastle Ottawa Scale for bias [34]. This was supported by R.H. The Newcastle Ottawa Scale [34] assigns points for selection, comparability, and exposure/outcome to a maximum of 9. A score of 0–3 is considered very high risk, 4 to 6 high risk, and ≥ 7 low risk. The authors chose not to exclude the few potentially biased studies from the review. However, all of these studies, which had high risk of bias by one or both reviewers (R.B., S.S.), were clearly noted in the text. Any disagreements in the risk of bias were discussed with another author.

### 2.4. Statistical Analysis

A formal meta‐analysis was not performed because of the small study numbers and heterogeneity of the studies in terms of study protocols and form of data. The results are presented descriptively, utilising simple statistical analysis and tabular and graphical representation, for a narrative synthesis.

## 3. Results

### 3.1. Quality of Studies

Nineteen studies were found pertaining to perioperative (30 days) and postoperative (up to 120 days) surgical outcomes in FMS. These were published from 1999 to 2024 [35–53]. The PRISMA flowchart [54] details the process of article selection and exclusion (Figure 2). The quality of studies was mostly good as shown in Table 1. Two studies, one by Gonzalez et al. [44] and one by Thorp and Ritzline [43] were deemed to have a very high likelihood of bias because of selection and outcome bias. As a meta‐analysis was not performed, these studies were still included in the review to allow for a complete picture of the literature. Of the 19 included studies, 6 assessed the extent of systemic FMS symptomatology using the 2011 FM survey criteria score [55]. This score forms the basis of the diagnostic criteria but does not dichotomise according to definitive FMS diagnosis [35–37, 41, 46, 53]. These six studies will be considered separately (Supporting Table 1b) but are included because of the general acceptance that FMS represents a continuum of symptomatology [19] and recent reviews have not made a distinction between these types of studies, and those that diagnose based on formal criteria [29, 30]. Four, out of the thirteen remaining studies, specifically analysed outcomes within the first 30 days postsurgery [39, 40, 42, 44]. The remaining nine studies *included* data on the first few months postsurgery relevant to the study questions [38, 43, 45, 47–52]. Studies were mostly cohort analyses with a few case control reports (Supporting Table 1a, 1b). There were no randomised controlled studies. We did not specifically conduct a sensitivity analysis with respect to study bias. However, as may be seen in Table 1, removing the studies with a high likelihood of bias did not result in a change in the outcomes. Complications for orthopaedic surgery are more often investigated, and this skewed the results in this regard.

**Table 1:** Study bias.



*Note:* The bias score is derived from the Newcastle Ottawa Scale [34]. All studies are labelled by author, and colour coded based on outcome. Here, red represents outcomes worse than the control population, yellow the same as, and green better than the control populations.

### 3.2. Speciality Spread

The majority of the FMS studies identified (12/19) were concerned with orthopaedic surgery [35–39, 44, 47–52]. The remaining seven studies were related to general surgery (cholecystectomy/diverticular surgery) [42, 45], gynaecology (hysterectomy/laser ablation) [41, 45], maxillofacial (temporomandibular joint [TMJ] surgery) [43]. While TMJ surgery could be considered orthopaedic in nature given its joint involvement, we have reported it separately as the surgical context differs from large joint orthopaedics, and TMJ dysfunction is often regarded as part of the FMS phenotype. Two studies, by Hesler et al. [40] and Larach et al. [46], analysed all surgery types.

### 3.3. Perioperative Outcomes Under 30 Days

#### 3.3.1. Pain Outcomes Under 30 Days

Costantini et al. [42] conducted a longitudinal study on the impact of laparoscopic cholecystectomy on pain and sensitization in female FMS patients aged 18–70. Five cohorts were analysed, including two groups undergoing surgery for symptomatic biliary calculosis: FMS (*n* = 31) and non‐FMS (*n* = 26). Comparisons were made with FMS patients not undergoing surgery, including those with symptomatic calculosis (*n* = 27), asymptomatic calculosis (*n* = 28) or no calculosis (*n* = 30). FMS diagnosis was confirmed using ACR 1990 and 2010 criteria, with baseline spontaneous pain scores ≥ 50/100 mm (VAS) and stable amitriptyline use.

In FMS patients, postoperative localised pain (VAS) was 5–10/100 mm higher than non‐FMS patients during the first postoperative week [42]. Global musculoskeletal pain and sensory thresholds worsened significantly at 1 week (*p* = 0.01), with partial recovery by 1 month and return to baseline by 3 months. At 6 and 12 months, spontaneous pain and sensory thresholds improved beyond baseline in FMS patients undergoing surgery (*p* < 0.05, *p* < 0.0001). Conversely, conservatively managed FMS patients with symptomatic calculosis showed progressive sensitisation, with significant increases in musculoskeletal pain and reduced muscle pain thresholds at 12 months (*p* < 0.05, *p* < 0.0001). The data, tentatively, suggest that untreated visceral pain contributes to sensitisation in FMS, while surgical intervention may alleviate global pain and improve sensory thresholds over time, although findings are muted by possible patient study selection bias [42].

Gonzalez et al. [44] investigated postoperative pain after knee arthroscopy in 13 FMS patients and 13 matched controls. FMS patients reported higher total pain scores (6.3 vs. 2.3/10, VAS) during the first postoperative week, though differentiation between site‐specific and global pain was not provided.

#### 3.3.2. Complication Outcomes Under 30 Days

Donnally et al. [39] analysed perioperative outcomes following posterior spinal fusion using data from the US Nationwide Medicare Database (2005–2014). Among 9304 FMS patients matched 1:1 with controls, 30 day complications included higher rates of acute postoperative haemorrhagic anaemia (OR 2.58; *p* < 0.001) and readmissions (RR 1.123; *p* = 0.007). The increased anaemia risk was attributed to serotonergic drug use, though transfusion rates were unchanged. FMS diagnoses were ICD‐coded but lacked details on diagnostic criteria or duration.

Hesler et al. [40] reviewed 21.78 million inpatient discharge records from seven US states to evaluate cardiovascular complications in FMS patients (*n* = 89,589). Matching based on demographics and procedural variables did not identify any increased cardiovascular risk. Surprisingly, in‐hospital mortality was slightly lower in FMS patients (OR 0.81, 99% CI: 0.73–0.89, *p* < 0.001). FMS diagnoses were inferred using ICD 729.1, limiting diagnostic specificity.

### 3.4. Perioperative Outcomes Over 30 Days

#### 3.4.1. Pain Outcomes Over 30 Days

Two studies examined pain outcomes beyond the 30‐day postoperative period, including one at 3 months and one at 6 months, which provide valuable context for the postsurgical course and include data from the immediate postoperative period [38, 42].

Ablin et al. [38] conducted a small, single‐centre study in Israel comparing outcomes in adult FMS patients (diagnosed by ACR 1990) and healthy controls (*n* = 11 FMS, *n* = 28 HC) following nonmalignant spinal surgery. They assessed WPI, SSS, preoperatively and at 3 months postsurgery. They found a marginal reduction in FMS patients’ WPI (−20.3%, from 6.7 to 5.4, *p* = 0.04) but no significant change in SSS (+3.6%, from 7.6 to 7.8). In contrast, controls showed significant reductions in both WPI (−42.9%, from 3.8 to 2.2, *p* < 0.001) and SSS (−50.1%, from 2.6 to 1.3, *p* < 0.01). These findings suggest that FMS patients experience less improvement in spontaneous pain and constitutional symptoms compared to controls, though relative changes from baseline may not fully reflect clinical significance. The study did not address surgical site pain or regional pain.

Costantini et al. [45] followed FMS patients (diagnosed by ACR 2010) with either endometriosis (*n* = 25) or diverticulosis (*n* = 24) before and after surgery. In the endometriosis group, laser treatment (*n* = 12) reduced painful menstrual cycles from 4.83 to 1.33 (*p* < 0.0006) and decreased noncyclic FMS flares from 12 to 7 (*p* < 0.003), as well as improving pressure pain thresholds at tender points (+20%, *p* < 0.02) and muscle pain thresholds (+10%, *p* < 0.0002). In diverticulosis, surgery (anterior sigmoid resection, *n* = 9) reduced acute abdominal pain episodes from 1.78 to 0.00 (*p* < 0.0003) and FMS flare frequency from 12 to 8 (*p* < 0.03) over 6 months. In addition, pressure pain thresholds at tender points increased (improved) by +20% (*p* < 0.04), and muscle pain thresholds improved by +20% (*p* < 0.05). These results support the notion that resolving visceral nociceptive pain can reduce overall pain and improve FMS sensory thresholds.

### 3.5. Operation‐Related Complications Over 30 Days

Five studies examined operation‐related complications extending beyond 30 days. Two studies by Costantini et al. [42, 45] found no increased complications in FMS patients following cholecystectomy, endometriosis laser therapy or diverticulosis surgery, though these studies were not designed to specifically assess complications.

Donnally et al. [39] reported higher pneumonia rates at 90 days (OR: 3.73, *p* < 0.0001) in FMS patients following spinal fusion, possibly attributed to inadequate analgesic control affecting respiratory effort. Moore et al. [47] found increased complication rates in FMS patients following total knee arthroplasty, including urinary tract infections, anaemia, transfusions, pneumonia and kidney failure (OR 1.95, *p* < 0.001). Morrell et al. [50] noted that FMS patients had increased opioid use, greater risk of dislocation (OR 1.9; *p* < 0.0001) and longer hospital stays after total hip replacement (9.4 vs. 2.9 days, *p* < 0.0001). Sanchez et al. [49] reported higher complication rates in FMS patients after total shoulder arthroplasty, including urinary tract infections (OR 4.49), pneumonia (OR 3.46), sepsis (OR 3.15), wound infection (OR 2.82) and cardiac events (OR 2.72), among others (*p* < 0.05). Similarly, Nelson et al. [51] found that FMS patients had higher odds of medical complications (OR 2.02; *p* < 0.001), implant‐related complications (OR 1.6; *p* < 0.001) and 90 day readmissions (12.5% vs 11.6%, OR 1.71; *p* < 0.001) following total hip replacement.

Qureshi et al. [48] identified that FMS patients were more likely to have ongoing narcotic prescriptions at 3 months following discectomy, with 25% of those with active prescriptions having FMS, compared to 15.7% in the nonactive group (*p* < 0.001). Sheth et al. [52] found that chronic pain and FMS were associated with continued opioid use at 90 days after total hip or knee arthroplasty, although FMS was not independently significant when controlling for preoperative opioid use.

### 3.6. Function Outcomes Between 30 and 120 Days

Four studies assessed functional outcomes beyond 30 days postsurgery [38, 43, 44]. Ablin et al. [38] found that FMS patients had less improvement in physical functioning, as measured by the SF‐36, following spinal laminectomy for chronic claudication or myelopathy. At 3 months, FMS patients showed no improvement in physical function (29.5) compared to controls (60.1, *p* < 0.001), who demonstrated significant improvement.

Gonzalez et al. [44] found that FMS patients had a longer rehabilitation period following knee arthroscopy, with a trend towards a slower resolution of symptoms (5.9 vs. 1.7 months). However, functional outcomes were reported as ‘good’ in 77% of FMS patients and 100% of controls, though no statistical analysis was performed.

Ablin et al. [38] studied TMJ correction surgery in a cohort of 4 FMS patients and 24 controls. No significant differences were observed in pain, mouth opening or dietary restrictions at 6 weeks postsurgery, though the small sample size limits the interpretability of these findings.

### 3.7. Studies Interrogating the 2011 FMS Survey Criteria Score

The 2011 FM survey criteria score [55], a composite of widespread pain and symptom severity and basis of the ACR Diagnostic Criteria [33], has been widely used to quantify FMS‐like features in surgical populations, revealing associations with pain and function. Several studies found that higher FM scores strongly correlated with greater preoperative pain intensity and symptom burden. For instance, Janda et al. [41] and Cheng et al. [35] reported significant positive correlations between FM scores and both surgical site and widespread pain before surgery. Brummett et al. [37] corroborated these findings, with higher FM scores linked to spontaneous perioperative pain at rest.

Postoperative outcomes presented a more varied picture. Janda et al. [41] and Brummett et al. [37] showed that patients with higher FM scores used more opioids postoperatively, even when accounting for preoperative opioid use, suggesting FM symptomatology might have amplified perioperative pain responses. Conversely, Cheng et al. [35] and Larach et al. [46] observed no significant postoperative pain or opioid differences in multivariate analyses, reflecting inconsistencies in FM score predictive power. Schrepf et al. [36] added nuance, highlighting an important subgroup of patients with persistently elevated FM scores 6 months after operative treatment (joint replacement), in spite of resolution of surgical pain, possibly pointing to central mechanisms.

Functional outcomes further emphasised nociplastic impacts. Schrepf et al. [36] and Cheng et al. [35] noted poorer physical recovery in patients with high FM scores, while Werkman et al. [53] found associations with jaw dysfunction, underscoring functional limitations beyond pain severity.

## 4. Discussion

**Figure 3:**
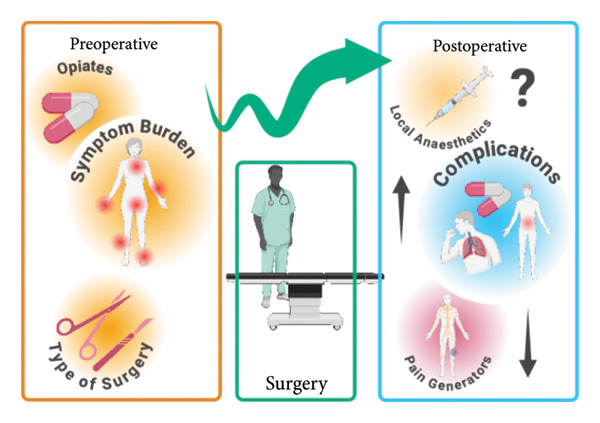
Overview of findings. Preoperatively factors such as the amount of symptom burden, opiate use and the type of surgery appear to predict more problems postsurgery. Following surgery, patients with fibromyalgia often experience more complications, though their pain generators may be reduced. There is little evidence to suggest whether local anaesthetics work differently in these patients. Figure created in BioRender (2025) https://BioRender.com/d19t952 (https://www.biorender.com).

This systematic review aimed to assess the perioperative experience of FMS patients undergoing surgery, focussing on postoperative pain, anaesthetic efficacy, complications and functional outcomes. The impetus for this review was the UK FMS guideline patient focus group, which highlighted gaps in understanding the specific perioperative challenges faced by this patient population. However, the evidence identified was sparse, particularly in the early postoperative period (up to 30 days), as illustrated in Supporting Table 1a, 1b. The results are summarised in Figure 3.

### 4.1. Pain Outcomes and LA Efficacy

The studies reviewed suggest that FMS patients experience worse postoperative pain compared to non‐FMS controls in the immediate period following surgery. Two studies indicated heightened surgical site pain and global musculoskeletal pain in FMS patients, who also exhibited reduced mechanical sensory thresholds, indicating an increase in generalised pain sensitivity after surgery [42, 45]. These findings underscore the heightened pain burden in FMS patients and the potential for enhanced pain perception in the postoperative setting. Studies assessing the FM survey score often found associations with increased postoperative opioid administration [37, 41], but this was not universal across all surgery types [35, 46]. Extrapolating these findings to a population of patients that would meet diagnostic criteria, however, one might expect this association to strengthen rather than weaken, a sentiment which aligns with earlier evidence that FMS patients experience more pain for surgical incision than controls [26].

Anecdotal reports from patients indicated delayed onset times for LAs, prompting an investigation into the pharmacological efficacy of LAs in this population. However, this review found limited evidence to refute altered LA kinetics in FMS patients, and certainly regional anaesthesia and neuraxial anaesthesia are still effective [35, 37] in those with fibromyalgia symptomatology. The phenomenon of wind‐up and central sensitisation in FMS likely contributes to the perception of postoperative pain, but further studies are needed to clarify whether the pharmacodynamics of LAs differ in FMS patients, particularly in terms of onset time and duration [57, 58].

### 4.2. Postoperative Complications

We also sought to identify complication rates up to 30 days. In this regard, two large cohort studies provided some data. Overall cardiovascular complications were unchanged in FMS [40] providing reassurance that risk factors of obesity and reduced movement often present in FMS [59, 60] will not translate into perioperative morbidity from cardiac causes.

However, studies assessing complications beyond 30 days corroborated existing literature, suggesting a higher rate of complications and increased healthcare utilisation in FMS patients across various surgical specialties, particularly orthopaedic procedures [39, 47, 49–51]. Notably, after spinal fusion surgery, higher rates of anaemia and readmissions were observed in FMS patients [39]. The reason for this anaemia is not clear though possible mechanisms could include nutritional deficiencies such as B12 [61], an exaggerated stress response affecting intraoperative haemodynamics or the use of medications (e.g. NSAIDS). Furthermore, pneumonia rates were significantly higher in FMS following spinal surgery, a finding consistent with theoretical considerations that surgeries that impair respiratory function cause hypoventilation and atelectasis from poor mucus clearance [39].

The review also suggested that FMS patients tend to experience prolonged opioid use after surgery. Qureshi et al. identified that FMS was associated with ongoing narcotic prescriptions at 3 months following discectomy, a finding that was potentially confounded by preoperative opioid use [48]. This association was similarly observed in other studies examining hip and knee arthroplasties [50, 52]. These findings suggest that preoperative symptom severity, as measured by FM survey criteria scores, may predict both higher baseline opioid use and a more significant increase in opioid requirements postoperatively [37, 41]. It should be noted that much of the opioid data comes from the USA which might bias the data because of prescribing practices.

### 4.3. Functional Outcomes

The review identified limited data on functional outcomes beyond 30 days postsurgery. Two studies found that FMS patients had poorer functional recovery compared to non‐FMS controls [38, 44] while a small study with limited statistical power found no difference in postoperative function [43]. Studies examining FM survey criteria scores revealed that patients with higher baseline symptomatology had worse postoperative physical function in the short term (e.g. within the first 2 days and at 6 months) [35, 36]. While these results are suggestive of poorer functional outcomes in FMS patients, further research is needed to more conclusively determine the long‐term impact of surgery on functional recovery in this population. The present review was not designed to evaluate long‐term outcomes, and thus, the findings on function are limited to the early postoperative period.

### 4.4. Surgically Amenable Pain Amplifiers

Findings from Costantini et al. are noteworthy. Their studies on visceral surgery suggest that surgery to remove visceral pain generators can lead to an improvement in widespread pain and sensory thresholds in FMS patients, in spite of an initial worsening of surgical site pain and spontaneous musculoskeletal pain [42, 45]. These results tentatively suggest that addressing peripheral pain sources may reduce generalised hypersensitivity in FMS. This notion is supported by similar findings in a cohort undergoing spinal surgery, where widespread pain improved, albeit to a lesser extent than in controls [38]. In addition, the theory of “bottom‐up” pain amplification, as proposed by Schrepf et al. [36], suggests that peripheral pain sources, such as musculoskeletal nociception, may contribute to generalised FMS pain. Coexistent FMS is common in patients with rheumatoid arthritis. While traditional treatments targeting inflammation, such as tumour necrosis factor‐alpha (TNF‐α) inhibitors, may reduce apparent disease activity, they are often insufficient to alleviate pain. Newer therapies, such as JAK‐STAT inhibitors, which act downstream to modulate proinflammatory signalling pathways, appear to offer greater benefits for pain control [62]. This discrepancy may support a “bottom‐up” model of pain in rheumatoid arthritis, whereby peripheral inflammatory mechanisms continue to drive symptoms because of incomplete control of proinflammatory mediators, not captured by standard inflammatory assays. However, further studies are needed to confirm whether surgical interventions that remove peripheral pain generators can lead to broader improvements in FMS pain and symptomatology, and why.

### 4.5. Comparison With Previous Literature

This review aligns with recent findings in the literature, which suggest that FMS is a significant risk factor for increased postoperative pain, worse functional outcomes, higher opioid administration and a higher incidence of medical and surgical complications following surgery [29, 60]. Our findings extend beyond orthopaedics to include a broader range of surgical specialties, particularly focussing on the early postoperative phase. While previous reviews primarily concentrated on long‐term outcomes, our findings emphasise the need for further exploration of the immediate postoperative course for FMS patients.

### 4.6. Mechanistic Insights

**Figure 4:**
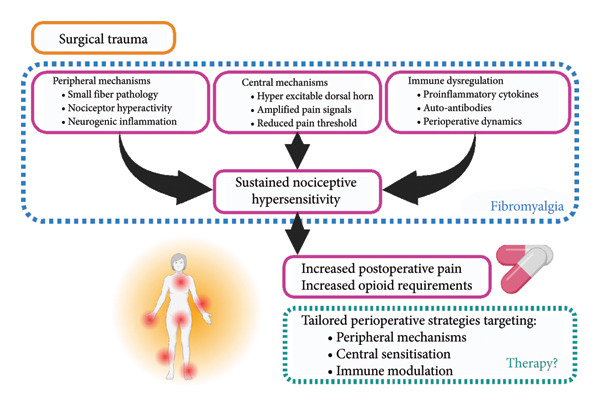
FMS mechanisms. Following surgical trauma, those with fibromyalgia may experience increased symptom burden via several mechanisms including peripheral, central and immune pathways. Potential peripheral strategies may include nutrition and medication review. Central sensitisation may be targeted through prehabilitation (aerobic and psychological), neuromodulation and intraoperative neuraxial techniques. Immune modulation could involve therapies such as plasma exchange. Figure created in BioRender (2025) https://BioRender.com/numg75a (https://www.biorender.com).

Emerging mechanistic insights into fibromyalgia suggest that pain may be underpinned by a convergence of central sensitisation [3, 8–10], peripheral nociceptor hyperactivity [5–7] and immune dysregulation [13–17]. In the context of postsurgical pain, these pathways may be additionally hijacked [63]. Central sensitisation, marked by heightened excitability of spinal and supraspinal neurons, lowers pain thresholds and amplifies nociceptive input, while peripheral contributions such as small fibre pathology and neurogenic inflammation may further exacerbate pain signalling. Nociceptive (inflammatory) and neurogenic pain from surgical insult could, unsurprisingly, be enhanced in this context. Concurrently, immune alterations, including elevated pro‐inflammatory cytokines [64] and pathogenic autoantibodies [13–15], may sustain nociceptive hypersensitivity. Although not presently understood, cytokine production in FMS postsurgery could be upregulated. To complicate matters further, some authors suggest that complex regional pain syndrome may mimic FMS postsurgery [65]. Together, these mechanisms align with clinical observations of increased postoperative pain and opioid requirements in fibromyalgia, and support the need for tailored perioperative strategies that target both central and peripheral contributors to nociplastic pain. Failure to adequately control perioperative pain in patients with FMS may contribute to worsening central sensitisation and symptom progression, and in some cases may be implicated in the development of new fibromyalgia‐like syndromes. This has been summarised in Figure 4.

## 5. Limitations

This review highlights several limitations in the current body of research, stemming from issues of design, diagnostic criteria and data quality. A major challenge is the heterogeneity across studies. Variations in surgical types, diagnostic criteria for FMS and outcome measures make cross‐comparison difficult and limit the generalisability of findings. In addition, there are important geographic differences in opioid prescribing practices and surgical customs. There may well be publications’ biases inherent in the data. Furthermore, most studies relied on retrospective data, which is prone to bias, confounding factors and limited ability to infer causality, underscoring the need for prospective cohort studies and randomised controlled trials.

Diagnostic ambiguity further complicates the evidence base. Database studies cannot interrogate how patients were diagnosed, potentially misclassifying patients. Others used FM survey criteria scores [35–37, 41, 46, 53] which, while useful, may not directly correlate with disease progression and can introduce bias when patients are categorised into low‐ and high‐symptom groups. In addition, inconsistent definitions of key outcomes like “surgical site pain” and “postoperative pain” may confuse interpretation and limit the applicability of findings.

Other limitations include small sample sizes, which reduce the statistical power to detect meaningful differences, especially in functional outcomes and rare complications. Bias and confounding factors, such as inadequate adjustment for variables like preoperative opioid use, further weaken the evidence base. Inconsistent reporting of baseline characteristics exacerbates these challenges.

## 6. Recommendations

Although further research is needed, we have compiled a set of clinical recommendations that integrate current evidence with best practice principles. These suggestions aim to support clinicians in optimising perioperative care for patients with fibromyalgia, while also highlighting key gaps in the existing research landscape. Table 2 summarises practical considerations across the surgical pathway. Recommendations have been made on the available information in the review; however, in the Supporting Figure S2, we have included a composite analysis to demonstrate how the FM survey criteria score data match with the FMS diagnostic study data.

**Table 2:** Clinical recommendations for perioperative management in fibromyalgia patients, *‘Berwick Criteria’*.

Preoperative	• Screen for FMS symptoms using tools like the FM Survey Score [54]
	• Optimise preoperative opioid use and centrally acting medications
	• Provide preoperative counselling and shared decision‐making around pain expectations (*e.g.* perhaps involving transitional pain service)
	• Engage preoperative coordination protocols between surgical, anaesthetic, perioperative care/recovery and potentially pain teams

Intraoperative	• Identify patients on surgical lists at team brief
	• Employ regional or neuraxial anaesthesia where appropriate
	• Use multimodal analgesia, prioritising non‐opioid and pre‐emptive agents

Postoperative	• Titrate opioids with a plan for weaning, alongside non‐opioid analgesia
	• Monitor for complications such as pneumonia and anaemia, especially after spinal or thoracic surgery
	• Encourage early mobilisation and pulmonary care

Rehabilitation & Follow‐Up	• Individualise rehabilitation plans addressing both physical and psychological needs
	• Monitor pain and function recovery; refer to pain services as needed
	• Intervene early if prolonged opioid use is observed

Systems & Research Priorities	• Standardise FMS diagnosis in surgical settings AND postoperative pain metrics
	• Incorporate FMS considerations into surgical audits and quality tracking
	• Conduct trials exploring tailored perioperative strategies for FMS patients (*e.g.* aerobic prehabilitation, nutritional optimisation or neuromodulation)
	• Look to assess the efficacy of local anaesthetics
	• Study the immune alterations perioperatively in FMS patients

## 7. Conclusions

The evidence on the immediate postoperative course for FMS patients remains limited, which is surprising given the high prevalence of the condition and the impact of surgery on pain sensitivity. There is some indication that greater FMS symptom severity may be a risk factor for poorer perioperative outcomes [37, 41, 42, 45]. Future research should focus on elucidating the mechanisms of heightened nociplastic pain responses in FMS postsurgery and the postoperative trajectory for FMS patients. Investigating tailored analgesic regimens, including nonopioid strategies and pre‐emptive multimodal approaches, could optimise postoperative pain management. Rehabilitation programs that address both physical and psychosocial recovery require further refinement to improve long‐term outcomes. In parallel, efforts are needed to standardise diagnostic and predictive tools, such as fibromyalgia scores, to enable stratified perioperative care. At present, the essential features of a peri‐surgical pathway for patients with fibromyalgia remain unclear. Potential components could include aerobic exercise [66] prehabilitation, structured medication review, nutrition and neuro‐modulatory therapies such as, repetitive transcranial magnetic stimulation [67], and transcranial direct cranial stimulation [68], which might aim symptom control perioperatively. Ultimately, progress in this field will depend on high‐quality randomised controlled trials with larger cohorts, standardised diagnostic criteria and longitudinal designs to better define surgical outcomes in fibromyalgia.

While some evidence suggests that FMS patients may experience worse postoperative pain and functional recovery, it is true that patients with nociplastic pain may also experience nociceptive pain amenable to treatment [11]. There may also be a dynamic interplay between these two, underscoring the need for careful discussions with patients. This insight warrants further investigation and will influence multidisciplinary decision‐making, in‐patient partnership and surgical interventions in FMS, as recommended by the RCP UK guidelines [69].

## Ethics Statement

No ethical approvals or patient consent were required for this study.

## Conflicts of Interest

Andreas Goebel receives consultancy fees from UCB. Richard J. Berwick receives consultancy fees from BioHaven and previously UCB. The other authors have no conflicting interests to declare.

## Author Contributions

Project conceptualisation was by Andreas Goebel and search design by Andreas Goebel and Michelle Maden. Ruaraidh Hill advised on methodology. The author team developed the (revised) protocol. Michelle Maden conducted the searches. Richard J. Berwick, Sara Siew and Sarah Curtis carried out the screening. Richard J. Berwick and Sara Siew extracted the data and Richard J. Berwick wrote the manuscript with support from Andreas Goebel and Ruaraidh Hill. All authors agreed on the final manuscript.

## Funding

Richard J. Berwick is supported by a Versus Arthritis Fellowship (22976) and by a PhD grant from the Pain Relief Foundation, UK.

## Supporting Information

Additional supporting information can be found online in the Supporting Information section.

## Supporting information


**Supporting Information 1** Figure 1: The search strategy employed. Figure 2: A sensitivity analysis comparing FMS diagnostics with survey scores for outcome measures.

**Supporting Information 2** Table S1: Outcomes of FMS diagnostic studies including bias scores.

**Supporting Information 3** Table S2: Outcomes of FM survey score studies including bias scores.

## Data Availability

Data sharing is not applicable for this article as no datasets were generated or analysed during the current study.
