# Supplementary material for: Pain in People With Fibromyalgia Syndrome (FMS) Undergoing or Following Surgery: A Systematic Narrative Review
Source: Pain Res Manag. 2026 Jan 22;2026:2352060. doi: 10.1155/prm/2352060 (PMC12824520; doi:10.1155/prm/2352060)
Supplement: Supplementary file 1 — Supporting Information 1 Figure 1: The search strategy employed. Figure 2: A sensitivity analysis comparing FMS diagnostics with survey scores for outcome measures. [file PRM-2026-2352060-s001.docx]

**Supplementary Material**

**Figure S1_Search Strategy Employed**

**MEDLINE**

**Ovid MEDLINE(R) ALL <1946 to June 28, 2024>**

1 Fibromyalgia/

2 fibromyalgi*.tw,kw.

3 fibrositis.tw,kw.

4 1 or 2 or 3

5 peri-operative*.tw,kw.

6 perioperative*.tw,kw.

7 pre-operative*.tw,kw.

8 preoperative*.tw,kw.

9 Postoperative Period/

10 Preoperative Period/

11 exp Perioperative Period/

12 exp surgical procedures, operative/

13 Postoperative Complications/

14 post-operative*.tw,kw.

15 postoperative*.tw,kw.

16 post-surg*.tw,kw.

17 postsurg*.tw,kw.

18 surger*.tw,kw.

19 surgical*.tw,kw.

20 operation*.tw,kw.

21 exp Pain, Postoperative/

22 or/5-21

23 4 and 22

24 limit 23 to (english language and yr="1990 -Current")

**CENTRAL Cochrane Central Register of Controlled Trials**

**Issue 2 of 12, June 28, 2024**

#1 MeSH descriptor: [Fibromyalgia] this term only

#2 (fibromyalgi*):ti,ab,kw

#3 (fibrositis):ti,ab,kw

#4 {OR #1-#3}

#5 (peri-operative*):ti,ab,kw

#6 (perioperative*):ti,ab,kw

#7 (pre-operative*):ti,ab,kw

#8 (preoperative*):ti,ab,kw

#9 MeSH descriptor: [Postoperative Period] this term only

#10 MeSH descriptor: [Preoperative Period] this term only

#11 MeSH descriptor: [Perioperative Period] explode all trees

#12 MeSH descriptor: [Surgical Procedures, Operative] explode all trees

#13 MeSH descriptor: [Postoperative Complications] this term only

#14 (post-operative*):ti,ab,kw

#15 (postoperative*):ti,ab,kw

#16 (post-surg*):ti,ab,kw

#17 (postsurg*):ti,ab,kw

#18 (surger*):ti,ab,kw

#19 (surgical*):ti,ab,kw

#20 (operation*):ti,ab,kw

#21 MeSH descriptor: [Pain, Postoperative] explode all trees

#22 {OR #5-#21}

#23 #4 AND #22

Notes:

1. Limited to 1990-
2. It is not possible to apply an English language limit to records in CENTRAL

**Embase**

1974 to 2024 June 28

Embase <1974 to 2024 June 28>

1 fibromyalgia/

2 fibromyalgi*.tw,kw.

3 fibrositis.tw,kw.

4 1 or 2 or 3

5 peri-operative*.tw,kw.

6 perioperative*.tw,kw.

7 pre-operative*.tw,kw.

8 preoperative*.tw,kw.

9 postoperative period/

10 preoperative period/

11 exp perioperative period/

12 exp surgery/

13 postoperative complication/

14 post-operative*.tw,kw.

15 postoperative*.tw,kw.

16 post-surg*.tw,kw.

17 postsurg*.tw,kw.

18 surger*.tw,kw.

19 surgical*.tw,kw.

20 operation*.tw,kw.

21 exp postoperative pain/

22 or/5-21

23 4 and 22

24 limit 23 to (english language and yr="1990 -Current")

25 limit 24 to (conference abstracts or medline)

26 24 not 25

Notes

1. Removes conference abstracts

**Figure S2_Sensitivity Analysis for Diagnosis**

Across the 5 key outcomes (<30d pain, <30d complications, >30d pain, >30d complications, and >30d function), a composite score has been calculated for studies assessing study groups based on FMS diagnoses (magenta) and those using FM Survey Score groupings (yellow). The composite score represents the sum of values from each study, where a positive study outcome was represented by 1, a neutral outcome by 0, and a negative outcome -1.
