# Supplementary material for: Pain in People With Fibromyalgia Syndrome (FMS) Undergoing or Following Surgery: A Systematic Narrative Review
Source: Pain Res Manag. 2026 Jan 22;2026:2352060. doi: 10.1155/prm/2352060 (PMC12824520; doi:10.1155/prm/2352060)
Supplement: Supplementary file 2 — Supporting Information 2 Table S1: Outcomes of FMS diagnostic studies including bias scores. [file PRM-2026-2352060-s002.docx]

| **Study** | **Participants (n FMS + HC)** | **Cohort & Design** | **Findings** | **Primary Outcomes** | **Limitations** |
| --- | --- | --- | --- | --- | --- |
| **Hesler *et al.*^40^** | 89,589 + 89,589  *ICD code 729.1 | USA, Database review, until hospital discharge | No increase in cardiovascular complications, but a small reduction in in-hospital mortality (OR 0.81, p<0.001). | Cardiovascular, complications, in-hospital mortality | Unable to account for confounding factors such as severity of disease. ICD code 729.1 is only indicative of FMS. |
| **Sheth *et al.*^52^** | 258  Medical record | USA, Prospective cohort, 90 days | Chronic pain and FMS were associated with continued opioid use at 90 days after total hip or knee arthroplasty, although FMS was not independently significant when controlling for preoperative opioid use. | Opioid use post-surgery, chronic pain | Single centre, small sample size, results may not be generalisable to broader populations. |
| **Moore *et al.*^47^** | 152,755 + 152,755  Medical record (PearlDiver) | USA, Database review, 90 days | FMS patients had significantly increased odds of medical complications after total knee arthroplasty (OR 1.95, p<0.001), including UTIs, pneumonia, haemorrhagic anaemia, transfusion and kidney failure. | Medical complications post-surgery (UTIs, pneumonia, kidney failure, etc.) | Database-based study, potentially incomplete data regarding comorbidities or disease severity. |
| **Morrell *et al*.^50^** | 8,326 + 157,273  Medical record (PearlDiver) | USA, Database review, 90 days | After total hip arthroplasty, FMS patients had a higher opioid use at 90 days (OR 1.3, p<0.0001), at least partly reflecting increased preoperative opioid use, longer length of stay (9.4 vs. 2.9 ± 3.7 days, p<0.0001) and more hip dislocation (OR 1.9; p<0.0001). | Opioid use, healthcare utilisation | No direct measurement of pain or other post-surgical recovery factors, potential biases in opioid use reporting. |
| **Nelson *et al.*^51^** | 76,103 + 76,103  Medical record  (PearlDiver) | USA, Retrospective Cohort, 90 days | FMS patients had higher odds of medical complications (OR 2.05; p<0.001), implant-related complications (OR 1.60, p<0.001), and 90-day readmission rates (OR 1.71; p<0.001). | Medical complications, implant-related complications, 90-day readmission rates | Potential limitations in matching controls to FMS patients for underlying health status. |
| **Gonzalez *et al.*^44^** | 13 + 13  ACR 1990 | Cuba, Retrospective Cohort, 5.9 months | FMS patients had higher post-op pain than controls (6.3 vs. 2.3 on a 10-point VAS), but no statistical testing was reported to confirm this. | Post-operative pain | No statistical testing to confirm significance, small sample size. |
| **Sanchez *et al.*^49^** | 7,738 + 30,770 | USA, Database review, Pearl Driver, 90 days | Patients were 1:4 matched with controls. Multivariate regression revealed that, at 90 days: urinary tract infection (OR 4.49); wound dehiscence (OR 3.63); pneumonia (OR3.46); emergency department visit (OR 3.45); sepsis (OR 3.15); surgical site infection (OR 2.82); cardiac events (OR 2.72); acute kidney injury (OR 2.65); deep vein thrombosis (OR 2.48); haematoma (OR 2.03); and pulmonary embolism (OR 2.01) (p<0.05 for each) were more common in FMS patients. | Post-surgical complications (UTIs, wound dehiscence, pneumonia, sepsis, etc.) | Large sample size, database-based study, potential underreporting of less severe complications. |
| **Ablin *et al.*^38^** | 11 + 28  ACR2010 | Israel, Cohort Study, 3 months | FMS patients saw less improvement in WPI and SSS than controls at 3 months post-surgery. Controls experienced highly significant reductions of both SSS and WPI (-50.1% and -42.9%) p<0.01), whilst FMS patients experienced no reduction of SSS symptoms or the AIMS and only a small reduction in WPI (-20.3%, p<0.05). At three months, FMS patients showed no improvement in physical function (29.5) compared to controls (60.1, p<0.001), who demonstrated significant improvement. | AIMS (Arthritis Impact Measures Scale)^a^ , WPI (Widespread Pain Index)^b^, SSS (Symptom Severity Score)^b^, SF-36 McGill Pain Questionnaire^c^ and | No randomisation, small sample size, potential selection bias in FMS patients. |
| **Donnally *et al.*^39^** | 9,304 + 9,304  ICD Code | USA, Database review, 3 months | FMS patients had higher postoperative complications, including acute post-haemorrhagic anaemia (OR 2.58) and readmission rates (p<0.007) at 30 days. At 90 days, pneumonia (OR 3.73; p<0.001) and anaemia (OR 2.79; p<0.001) were more common. | Postoperative complications (haemorrhagic anaemia, readmission rates) | Database limitations, lack of control over preoperative conditions or surgical approaches. |
| **Qureshi *et al*.^48^** | 1321 discectomy patients + no HCs Medical record (PearlDiver) | USA, Database review, 90 days | FMS patients had higher odds of ongoing narcotic prescriptions at 3 months post-surgery (25.0% vs. 15.7%, p<0.001). | Ongoing opioid prescriptions after discectomy | Database-based study, may not account for confounders e.g. preoperative pain or opioid use. |
| **Costantini *et al.*^42^** | 116 + 26  ACR 1990 | Italy, Cohort Study, 12 months | FMS patients showed increased postoperative pain and worsened sensory thresholds, with gradual recovery to baseline by 3 months followed by improvement on baseline. | Postoperative pain, sensory thresholds | Small sample size, limited to a specific surgery (laparoscopic cholecystectomy). Selection bias. |
| **Costantini *et al.*^45^** | 142 + 0  ACR 2010 | Italy, Cohort Study, 6 months | Surgery led to improved pain and sensory thresholds in FMS patients, with reduced FMS flare frequency and pain intensity. In the endometriosis group, laser treatment (n=12) reduced painful menstrual cycles from 4.83 to 1.33 (p<0.0006) and decreased non-cyclic FMS flares from 12 to 7 (p<0.003), as well as improving pressure pain thresholds at tender points (+20%, p<0.02) and muscle pain thresholds (+10%, p<0.0002). In diverticulosis, surgery (anterior sigmoid resection, n=9) acute abdominal pain episodes from 1.78 to 0.00 (p<0.0003) and FMS flare frequency from 12 to 8 (p<0.03). Additionally, pressure pain thresholds at tender points increased by +20% (p<0.04), and muscle pain thresholds improved by +20% (p<0.05). | Pain levels, sensory thresholds, FMS flare frequency | Small sample size, limited to endometriosis and diverticulosis surgery. |
| **Thorp *et al.*^43^** | 4 + 24  Self-report | USA, Single Centre Cohort, 5 months | No difference was found in pain, mouth opening, or dietary restriction at six weeks post-surgery in FMS patients compared to controls. | Pain, mouth opening, dietary restriction post-TMJ surgery | Very small sample size (4 FMS vs. 24 controls). |
| *Abbreviations: FMS - fibromyalgia syndrome; HC - healthy controls; TMJ - temporomandibular joint*  **non-specific FMS diagnostic code.* | | | | | |

**Additional References**

a. Wolfe F, Clauw DJ, Fitzcharles MA, Goldenberg DL, Katz RS, Mease P, Russell AS, Russell IJ, Winfield JB, Yunus MB. The American College of Rheumatology preliminary diagnostic criteria for fibromyalgia and measurement of symptom severity. Arthritis Care Res (Hoboken). 2010;62:600-610.

b. Meenan RF, Gertman PM, Mason JH, Dunaif R. The arthritis impact measurement scales. Further investigations of a health status measure. Arthritis Rheum. 1982;25:1048-1053.

c. Hoffman DL, Dukes EM. The health status burden of people with fibromyalgia: a review of studies that assessed health status with the SF-36 or the SF-12. Int J Clin Pract. 2008;62:115-126.
