# Supplementary material for: Pain in People With Fibromyalgia Syndrome (FMS) Undergoing or Following Surgery: A Systematic Narrative Review
Source: Pain Res Manag. 2026 Jan 22;2026:2352060. doi: 10.1155/prm/2352060 (PMC12824520; doi:10.1155/prm/2352060)
Supplement: Supplementary file 3 — Supporting Information 3 Table S2: Outcomes of FM survey score studies including bias scores. [file PRM-2026-2352060-s003.docx]

**Table 1b – Chart of Study Outcomes – FM Survey Score Criteria**

These studies looked at 2011 FM Survey Criteria rather than using a strict FMS diagnosis and so many of these patients would not have met the diagnostic criteria necessarily.

| **Study** | **Cohort & Design** | **Findings** | **Key Measures** | **Limitations** |
| --- | --- | --- | --- | --- |
| **Brummett *et al.* (2014)^37^** | Prospective, US single-centre, hip and knee replacement patients (n=519), 6 months. | Higher FM scores were linked to greater pre-op opioid use and increased post-op opioid needs in opioid-naive patients. (p=0.0024). Neuraxial anaesthesia reduced opioid requirement (71.7mg; p<0.00001). | FM Survey Criteria^a^, opioid consumption; pain severity; neuraxial anaesthesia | No direct postoperative pain outcomes, limited to preoperative pain and opioid use |
| **Cheng *et al.* (2015)^35^** | Prospective, US single-centre, shoulder arthroscopy (n=92), 14 days. | Higher FM Survey Criteria scores were associated with increased preoperative pain and postoperative opioid use at 14 days. FM scores were not correlated with post-op pain or opioid use but predicted poorer recovery scores (p=0.001). | FM Survey Criteria^a^ opioid administration; pain severity (VAS); neuropathic pain (PainDETECT^b^); PROMIS Physical Function Score^c^; anaesthesia recovery (Quality of Recovery-9 Score^d^) | Differing baseline opioid use in groups, limited 14-day follow-up |
| **Janda *et al.* (2015)^41^** | Prospective, US single-centre, hysterectomy patients (n=195), until discharge. | Higher FM Survey Criteria scores were linked to more widespread and surgical site preoperative pain(p<0.0001). Multivariate regression confirmed association with postoperative opioid use controlling for preoperative use (p<0.0001). | FM Survey Criteria^a^; opioid administration; preoperative pain intensity (Brief Pain Inventory^e^) | Potential confounding by preoperative opioid use, small number meeting FMS criteria |
| **Larach *et al.* (2015)^46^** | Prospective, US single-centre, total knee arthroplasty, hip replacement, hysterectomy, thoracic surgery (n=913), 30 days. | FM Survey Criteria scores were positively correlated with postoperative opioid use in some surgeries. Post-op opioid use correlated with FM score in most surgeries, though hip replacement showed a negative correlation in multivariate analysis. | FM Survey Criteria^a^; opioid administration) | Limited to specific surgical types, multivariate results inconsistent |
| **Schrepf *et al.* (2016)^36^** | Prospective, US single-centre, hip and knee replacement (n=150), 6 months. | Most patients improved in widespread pain and constitutional symptoms by one-month post-surgery. However, by six months, 32% (n=48, p=0.004) had no improvement in FM scores, with a deterioration to pre-surgical levels, despite resolving acute surgical site pain. Surgical site pain was less common in the FM score “improved group” (n=102)* at one month (57% vs. 87%; p<0.001) and three months (27% vs. 46%; p=0.03) but was similar between groups at six months (26% vs. 31%). Physical function also improved more in the FM score improved group (p=0.021, n=102). | FM Survey Criteria^a^; pain severity; PROMIS Physical Function Score^c^ | High attrition rate, only 5/150 patients met FMS diagnostic criteria |
| **Werkman *et al.* (2021)^53^** | Retrospective cohort, US single-centre, TMJ arthroscopy (n=28), 1.6 months. | FM Survey Criteria scores not correlated with postoperative pain (VAS) but was associated with mobility score in the Jaw Function Limitation Score (p<0.05). | FM Survey Criteria score^a^; pain severity (VAS); Jaw Function Limitation Score^f^ | Small sample size, limited follow-up period |
| *Abbreviations: PROMIS - Patient-Reported Outcomes Measurement Information System; TMJ - temporomandibular joint; VAS - visual analogue scale. The FM Survey Criteria score is a self-report tool assessing widespread pain and somatic symptoms. *The FM Survey Criteria score “improved group” was defined as the top tertile of patients experiencing the most improvement in FM symptoms at 6 months.* | | | | |

**Additional References**

a. Wolfe F, Clauw DJ, Fitzcharles M-A, Goldenberg DL, Häuser W, Katz RS, Mease P, Russell AS, Russell IJ, Winfield JB. Fibromyalgia criteria and severity scales for clinical and epidemiological studies: a modification of the ACR Preliminary Diagnostic Criteria for Fibromyalgia. Journal of Rheumatology. 2011;38:1113-1122.

b. Freynhagen R, Baron R, Gockel U, Tolle TR. painDETECT: a new screening questionnaire to identify neuropathic components in patients with back pain. Curr Med Res Opin. 2006;22:1911-1920.PainDETECT1)

c. Cella D, Yount S, Rothrock N, Gershon R, Cook K, Reeve B, Ader D, Fries JF, Bruce B, Rose M, Group PC. The Patient-Reported Outcomes Measurement Information System (PROMIS): progress of an NIH Roadmap cooperative group during its first two years. Med Care. 2007;45:S3-S11.

d. Myles PS, Hunt JO, Nightingale CE, Fletcher H, Beh T, Tanil D, Nagy A, Rubinstein A, Ponsford JL. Development and psychometric testing of a quality of recovery score after general anesthesia and surgery in adults. Anesth Analg. 1999;88:83-90.

e Tan G, Jensen MP, Thornby JI, Shanti BF. Validation of the Brief Pain Inventory for chronic nonmalignant pain. J Pain. 2004;5:133-137.

f. Ohrbach R, Larsson P, List T. The jaw functional limitation scale: development, reliability, and validity of 8-item and 20-item versions. J Orofac Pain. 2008;22:219-230.
